# Syphilitic Hepatitis: A Rare Cause of Elevated Liver Function Tests

**DOI:** 10.7759/cureus.34312

**Published:** 2023-01-28

**Authors:** Shaharyar Salim, Rabeea Farhan, Asif Surani

**Affiliations:** 1 Department of Internal Medicine, Aga Khan University Hospital, Karachi, PAK; 2 Department of Environmental and Occupational Health, Rutgers School of Public Health, Rutgers University, Piscataway, USA; 3 Department of Internal Medicine, Medical College of Wisconsin, Milwaukee, USA

**Keywords:** treponema pallidum, hepatic enzymes, hepatic manifestations, sexually transmitted infection (sti), infectious hepatitis, liver function test (lft), sexual transmitted diseases, deranged liver function test, syphilis

## Abstract

Syphilitic hepatitis is a rare manifestation of syphilis with an incidence of 0.2-38%. We describe a case of a healthy, immunocompetent male patient with elevated liver function tests (LFTs) who was found to have syphilitic hepatitis.

A 28-year-old male with no past medical history presented with abdominal pain for two to three weeks. He also reported diminished appetite, intermittent chills, weight loss, and fatigue. His history was positive for high-risk sexual behavior including multiple partners and absence of using protection. His physical examination was remarkable for right-sided abdominal tenderness and a painless chancre on his penile shaft. His workup revealed elevated aspartate aminotransferase (AST: 169 U/L), alanine transaminase (ALT: 271 U/L), and alkaline phosphatase (ALP: 377 U/L). His abdominal CT scan was unremarkable except for the abdominal and pelvic lymphadenopathy. A thorough serology panel revealed negative hepatitis A, B, C, human immunodeficiency virus (HIV) (including HIV RNA), Epstein-Barr virus (EBV), and cytomegalovirus (CMV). His immunological workup was negative as well. His rapid plasma reagin (RPR) was reactive with positive IgG/IgM treponemal antibodies. He was managed as secondary syphilis and received 2.4 million units of benzathine penicillin. Upon follow-up after one week, he reported a complete resolution of his symptoms, and his LFTs were normalized on a repeat checkup.

Given the significant morbidity associated with a missed diagnosis, syphilitic hepatitis should be considered an essential part of the workup for evaluating elevated LFTs in an appropriate clinical setting. This case also highlights the importance of obtaining a comprehensive sexual history and performing a thorough genital examination.

## Introduction

Syphilis, commonly known as "the great imitator," can virtually affect any organ of the body. Syphilitic hepatitis, also called "luetic jaundice," is a rare manifestation of syphilis signifying spirochete dissemination to the liver. It was first recognized in 1585 [1] and was initially reported in the literature by Harn in 1943 [2]. The actual incidence of syphilitic hepatitis is uncertain; however, it occurs in 0.2-38% of patients with a history of syphilis diagnosis [3,4].

Syphilitic hepatitis is usually defined as a cholestatic pattern of liver enzyme elevation with serological treponemal evidence in the absence of other causes of hepatic dysfunction, and improvement after appropriate antimicrobial therapy [5]. Liver involvement can occur at any disease stage, although most cases have been reported to occur during the early stages of syphilis [6].

Due to the recent increase in the incidence of primary and secondary syphilis, it is imperative that clinicians should consider syphilis in the differential diagnosis of patients with liver dysfunction and elevated liver function tests (LFTs) of unclear etiology [5]. In this article, we describe a case of a healthy, immunocompetent 28-year-old male with elevated LFTs who was diagnosed with syphilitic hepatitis.

## Case presentation

A 28-year-old male with no significant past medical history presented to the emergency department with complaints of abdominal pain for two to three weeks. His abdominal pain was located in the right upper quadrant, was constant, radiated to the back, and throbbing in nature. His pain was aggravated by movements and deep breaths, with no significant relieving factors. He reported diminished appetite, chills, 10 pounds of weight loss, and fatigue which preceded his current symptoms by several months. Of note, the patient had a history of gonorrhea one year prior to this presentation and was treated appropriately. He reported being sexually active with his current female partner for more than three years. On enquiring about safe sexual practices, he admitted to have had unprotected sexual intercourse with other female partners. His recent rapid plasma reagin (RPR) screen was negative six months prior to the presentation. Interestingly, he had an urgent care visit one month prior to the presentation for a non-healing penile lesion; *Treponema pallidum* total antibody was negative at that time. He denied any history of drug abuse except for the minimal alcohol intake of one to two drinks of wine per week.

His physical examination was significant for temporal wasting and right upper quadrant abdominal tenderness without guarding or rigidity. A painless chancre was noted on the ventral aspect of the penile shaft without any obvious discharge. His blood work was remarkable for deranged LFTs - aspartate aminotransferase (AST) 169 U/L (peak: 197 U/L), alanine aminotransferase (ALT) 271 U/L (peak: 371 U/L), alkaline phosphatase (ALP) 377 U/L (peak: 529 U/L), bilirubin 1.0 mg/dL (peak: 2.4 mg/dL) with direct bilirubin of 0.6 mg/dL, and normal albumin and international normalized ratio (INR). The abdominal ultrasound and CT scan were unremarkable except for several large abdominal and pelvic lymph nodes.

During the hospital course, he underwent extensive workup for his elevated LFTs that revealed normal acetaminophen levels, negative hepatitis panel (A, B, and C), human immunodeficiency virus (HIV) screen and HIV-1 RNA, Epstein-Barr virus (EBV), and cytomegalovirus (CMV) (IgG and IgM) titers, anti-nuclear antibody (ANA) and anti-smooth muscle antibody (ASMA). His *Helicobacter pylori *(*H. pylori*) stool antigen was positive. In addition, he had a positive RPR screen with titers of 32 dils along with a positive treponema IgM/IgG antibody.

Due to the presence of a penile chancre, positive RPR screen and titers, and significant abdominopelvic lymphadenopathy, he was managed as secondary syphilis with intramuscular penicillin G benzathine 2.4 million units. He was also started on omeprazole, clarithromycin, and amoxicillin triple therapy for *H. pylori* eradication. His pain was managed with opiates which were tapered down during the hospital course. Though his LFT trend slightly worsened after penicillin administration, it subsequently improved with repeat testing (Table 1). Due to the overall stability and a slight improvement observed in the LFT trend, the liver biopsy was deferred.

**Table 1:** LFTs trend on presentation and after receiving antibiotics. LFT: liver function test

Variables	On presentation	24 hours after penicillin administration	72 hours after penicillin administration	Upon follow-up visit (2 weeks)
Alkaline phosphatase (44-147 U/L)	377 U/L	529 U/L	490 U/L	277 U/L
Aspartate aminotransferase (8-33 U/L)	169 U/L	197 U/L	152 U/L	32 U/L
Alanine aminotransferase (4-36 U/L)	271 U/L	371 U/L	326 U/L	83 U/L
Total bilirubin (0.1-1.2 mg/dL)	1.3 mg/dL	1.8 mg/dL	1.2 mg/dL	0.3 mg/dL
Direct bilirubin (<0.3 mg/dL)	0.6 mg/dL	0.6 mg/dL	0.4 mg/dL	-
Albumin (3.4-5.4 g/dL)	4.0 g/dL	3.9 g/dL	4.0 g/dL	4.2 g/dL
Gamma-glutamyl transpeptidase (5-40 U/L)	411 U/L	-	-	-

On the follow-up visit after two weeks, he reported improvement in his abdominal pain. His penile chancre was resolved and his LFTs improved significantly (Table 1). However, he was noted to have persistent inguinal lymphadenopathy.

## Discussion

We report a case of a healthy, immunocompetent 28-year-old male patient who presented with abdominal pain and deranged LFTs along with abdominopelvic lymphadenopathy. He was diagnosed with secondary syphilis and syphilitic hepatitis. Although there are no established criteria for the diagnosis of syphilitic hepatitis, Mullick et al. proposed the following criteria: (1) elevated liver enzymes indicating liver involvement; (2) positive serological evidence for syphilis; (3) exclusion of alternative causes of liver injury; and (4) improvement in liver enzyme after appropriate antimicrobial therapy [5]. Our patient satisfied all four diagnostic criteria.

Clinical signs and symptoms of syphilitic hepatitis can be non-specific. In a literature review of 97 cases by Huang et al., rashes were the most common clinical manifestation, followed by fatigue or poor appetite, fever, weight loss, and abdominal pain [6]. Common physical examination findings highlighted in the same review were hepatomegaly, lymphadenopathy, splenomegaly, and uveitis. Our patient had abdominal pain, weight loss, poor appetite, and fatigue along with a physical examination finding of lymphadenopathy which were similar to the aforementioned literature review.

The pattern of liver function testing in syphilitic hepatitis is typically cholestatic. However, hepatocellular or mixed patterns are also observed. Marked increase in ALP and gamma-glutamyl transferase (GGT) is also characteristic [6,7]. Our patient had a similar pattern of ALP and GGT elevation along with mild elevation of transaminases (ALT>AST). Intriguingly, our patient had worsening of ALP, bilirubin, and transaminases 24 hours after penicillin administration. It slightly improved after 72 hours but remained elevated compared to the pre-antibiotic levels. Elevated transaminases and bilirubin levels completely resolved after two weeks; ALP did down-trend but remained above the normal limits at the two-week follow-up visit. The worsening of LFTs after penicillin administration contrasts with the reported finding of Pereira et al. who noted significant improvement in transaminases and ALP at the 72-hour mark [8].

As stated above, our patient had a recent urgent care visit for a non-healing penile lesion. His *Treponema pallidum *total antibodies were negative at that time. This false negative could be due to prozone phenomena that result from overwhelming antibody titers which hamper the formation of antigen-antibody lattice required for the visualization of a positive flocculation test. The prozone effect has been previously reported in secondary syphilis leading to delayed diagnosis [9].

Interestingly, our patient had a positive concomitant *H. pylori *infection. Human studies have highlighted an association between *H. pylori *infection and disease progression in established chronic viral hepatitis (hepatitis B and C) [10]. Salehi et al. in the same article have also suggested a role of *H. pylori *infection in some cases of mild unexplained hypertransaminasemia with improvement after receiving eradication therapy [10]. Though our patient had positive *H. pylori *testing, he had a negative viral hepatitis panel including hepatitis B and C. In addition, the presence of penile chancre, positive serology of syphilis, and lymphadenopathy suggest syphilis be the likely etiology of LFT derangement in our patient.

## Conclusions

Syphilitic hepatitis is an important and under-recognized cause of LFTs elevation. Due to an increased incidence of syphilis, clinicians should consider this diagnosis in a patient with elevated LFTs of unknown etiology and high-risk sexual behavior. This report highlights the paramount importance of obtaining a comprehensive sexual history and performing a detailed genital examination. Timely diagnosis and prompt treatment with penicillin can lead to clinical recovery and prevention of complications.
